# Primary assessment of medicines for expected migrastatic potential with holographic incoherent quantitative phase imaging

**DOI:** 10.1364/BOE.488630

**Published:** 2023-05-16

**Authors:** Markéta Šuráňová, Miroslav Ďuriš, Irena Štenglová Netíková, Jan Brábek, Tomáš Horák, Veronika Jůzová, Radim Chmelík, Pavel Veselý

**Affiliations:** 1 Institute of Physical Engineering (IPE), Faculty of Mechanical Engineering, Brno University of Technology, Brno, Czech Republic; 2 CEITEC - Central European Institute of Technology, Brno University of Technology, Brno, Czech Republic; 3 General University Hospital in Prague, Department of Clinical Pharmacology and Pharmacy, Prague, Czech Republic; 4 Department of Cell Biology, and Biotechnology and Biomedicine Center of the Academy of Sciences and Charles University in Vestec (BIOCEV), Laboratory of Cancer Cell Invasion, Charles University, Prague, Czech Republic

## Abstract

Solid tumor metastases cause most cancer-related deaths. The prevention of their occurrence misses suitable anti-metastases medicines newly labeled as migrastatics. The first indication of migrastatics potential is based on an inhibition of *in vitro* enhanced migration of tumor cell lines. Therefore, we decided to develop a rapid test for qualifying the expected migrastatic potential of some drugs for repurposing. The chosen Q-PHASE holographic microscope provides reliable multifield time-lapse recording and simultaneous analysis of the cell morphology, migration, and growth. The results of the pilot assessment of the migrastatic potential exerted by the chosen medicines on selected cell lines are presented.

## 1. Introduction

The spread of cancer cells from a place of the original primary solid tumor is called metastasis [1] and accounts for more than 90% of oncological patient mortality [2,3]. Although it is a key cause of solid tumor treatment failure and mortality, metastasis remains poorly understood [3].

Generally, metastasis is a complex phenomenon that necessitates the use of multiple therapeutic agents to effectively prevent it from occurring [1]. Therefore, adopting a combination therapy model and targeting multiple pathways simultaneously appears to be the key to combating significant genomic and phenotypic alterations of metastatic tumor cells [4].

Solid tumor treatment should therefore be supplemented with drugs that inhibit the ability of tumor cells to penetrate through the extracellular matrix (ECM) and form metastases. Since the mechanisms determining clonal proliferation and cell migration are distinct, then the antiproliferative strategies in antitumor drug discovery should be complemented with those targeting mechanisms related to motility and migration [3].

However, in antitumor therapies a specific category of anti-invasive and anti-metastatic drugs had been missing until recently when the term “migrastatic” (from Latin “migrare” and Greek “statikos”) for drugs interfering with all modes of tumor cell migration and metastasis was proposed [3,5,6,7]. The formation of metastases is related to the metastatic cascade which describes the process by which invasive tumor cells leave the primary tumor, travel through the bloodstream, and eventually reach distant organs where one or more metastases form [3,8]. Therefore, migrastatics differ from traditional cytostatic drugs that primarily target cell proliferation (a process of cell growth and subsequent division, producing two daughter cells), as they interfere with all modes of tumor cell invasion and metastasis. To be accurate, migrastatics are a unique class of drugs that target local invasion and inhibit extravasation and metastatic colonization. This sets them apart from conventional cytostatic drugs that primarily aim to hinder only cell proliferation [3,9]. It is important to note that the development of efficacious migrastatics has been hindered by the absence of adequate detection methods. Using animal models for such research has been impractical due to the high costs and short animal lifespan. However, establishing sophisticated 3D culture systems has facilitated large-scale experimentation, offering a promising alternative [3].

To effectively realize the concept of migrastatics, two key requirements must be met. Firstly, there is a need to fine-tune regulations for the approval of antitumor drugs, emphasizing the antimetastatic effects claimed for migrastatics. This will allow a clinical evaluation of drug candidates even in the absence of tumor shrinkage [3]. Secondly, screening compound libraries should be allowed on a large scale and should particularly exploit the repurposing of extensively proof-safe medicines with expected migrastatic potential as well as finding novel compounds that exhibit low toxicity and interfere with tumor cell migration. Even though the tumor cell migration patterns can be different in 2D and 3D environments, initial evaluation of the drug’s potential to inhibit tumor cell migration in a simple 2D environment provides valuable indicators for follow-up invasion study in artificial 3D systems or, ideally, animal models [3].

In this article, we propose a 2D methodology based on digital holographic microscopy providing a quick and economically viable assessment of the drug's migrastatic potential. As our methodology involves imaging of tumor cells in simplified 2D environments and its outputs need further validation in more complex culture systems, we call our method the Primary Assessment of Migrastatic Potential (PAMP). Our method may also serve as the first step in considering particular migrastatic agents for the oncological treatment process. We show that migrastatic candidates can be quickly evaluated based on the cell dynamics of migration, morphological changes, cell growth, and cytopathogenicity. We do this by quasi-real-time monitoring of the cells. This helps us to also observe unexpected changes in the behavior of tumor cells towards unwanted increased directionality of random migration after the application of migrastatics.

To achieve this, we took advantage of the Quantitative Phase Imaging (QPI) [10,11] provided by the Coherence-Controlled Holographic Microscopy (CCHM). The CCHM [12–15], commercially available as a Q-PHASE microscope (manufactured by TELIGHT Ltd., Brno), makes it possible to quantitatively obtain the amplitude and phase retardation of the light wave transmitted through a sample. The reconstructed phase image carries quantitative information on the mass topography. This allows us to obtain morphology of cells in a marker-free (non-toxic) non-invasive way with nanometer axial resolution [13]. In biological applications, cell dynamics and morphology analyses, such as monitoring of the distribution of dry matter in cells [16–18] or cell tracking can be performed. The use of a low-coherence light source in this system enables the suppression of coherent noise, improved lateral resolution, and provides an optical sectioning effect using coherence gating [13]. Compared to other digital holographic microscopes that utilize coherent illumination, the coherence-gating effect provides us with QPI of a significantly higher phase quality in 2D as well as 3D culture conditions. This is crucial to the PAMP methodology proposed in this work as the higher signal-to-noise ratio and the increased spatial resolution allow for more robust cell segmentation and tracking of cell position. The practical advantage of the Q-PHASE microscope is the possibility of using multiwell observation chambers that provide us with higher throughput due to multifield time-lapse recording. This recording produces videos that allow us to make simultaneous comparisons of several drugs under the same culture conditions intelligible.

To validate the 2D proposed PAMP methodology, this pilot study analyzes the interaction of seven selected migrastatic candidates: 4-hydroxyacetophenone, belumosudil, doxycycline, fasudil, midostaurin, niclosamide, and pimozide with three human cell lines. These cell lines were chosen for this methodology: Normal human dermal fibroblasts (NHDF) and non-small lung cancer cells (A549) showing amoeboid motility in 3D collagen and invasive fibrosarcoma cell line (HT1080) showing mesenchymal motility in 3D collagen. We proceeded from the theory that individual tumor cells exhibit two interconvertible migration methods during tissue invasion, namely the mesenchymal and the amoeboid motility. Therefore, representatives of both groups were selected. The mesenchymal motility mode is characterized by elongated cell morphology, adherence to the surrounding extracellular matrix mediated by integrins, and degradation of the extracellular matrix by proteases. In contrast, during the amoeboid motility, cells are highly deformable, their adhesion to the extracellular matrix is rather weak, and proteolytic activity is reduced or absent [19,20]. Furthermore, we concluded from the studies that the characteristics of mesenchymal motility are not limited to individual migrating cells. Amoeboid cells are more flexible and can squeeze into small gaps to find their way but are unable to degrade the extracellular matrix or create new pathways. However, amoeboid motility can be as efficient as mesenchymal motility. In the presence of proteolytic inhibitors, mesenchymal cells spontaneously change to an amoeboid form, thus retaining the ability to migrate. Amoeboid motility was also found to be preferred in situations of metabolic stress, such as hypoxia, which may imply that the amoeboid motility mode is less energetically demanding or more efficient compared to the mesenchymal mode [19–23].

## 2. Materials & methods

### 2.1 Cell cultures

The A549 epithelium of human lung alveolar basal epithelial cells belong to the group of non-small cell lung cancer cells (NSCLC). The heterogeneous class of these tumors accounts for approximately 85% of all new diagnoses of lung tumor [24]. The human fibrosarcoma HT1080 cell line is a type of tumor that is aggressive with many local recurrences as well as lymph and parenchymal metastases. Survival in adults with fibrosarcomas is < 70% at two years and < 55% at five years [25–27]. The NDHF cell line of normal dermal human fibroblasts (gift of Dr. Eva Pagáčová, Institute of Biophysics, Brno, Academy of Sciences of the Czech Republic) plays an important role in the cell renewal system and in maintaining skin integrity [28].

Cells were grown at 37 ° C in a humidified incubator with 3.5% CO_2_ in Hank's Minimum Essential Medium (HMEM) supplemented with non-essential amino acids, 1 mM sodium pyruvate, 2 mM L-glutamine, 10% fetal bovine serum and gentamicin (10 µg/ml). For the time-lapse recording, the medium was enriched with 20 mM HEPES to maintain pH 7.4. A temperature of 37 °C was maintained in the environmental enclosure of the Q-PHASE microscope.

### 2.2 Migrastatic candidates

We compared the results with other researchers, for example, 4-hydroxyacetophenone (4-HAP) is known as a bioactive compound found in several medicinal herbs, exerts a potent stimulatory effect on hepatic bile secretion [29]. This chemical inhibits the adhesion and migration of tumor cells *in vitro* and reduces metastatic burden in an *in vivo* liver metastasis model [30].

Belumosudil (BEL) is a Rho-associated coiled-coil-containing protein kinase (ROCK) inhibitor that has been developed by Kadmon Pharmaceuticals for the treatment of chronic graft-versus-host disease (cGVHD) and systemic sclerosis (scleroderma). In July 2021, BEL received its first approval in the USA for the treatment of adult and paediatric patients aged ≥ 12 years with cGVHD after failure of at least two prior lines of systemic therapy. BEL is under regulatory review in Australia, Canada, the UK, and Switzerland for cGVHD. Clinical trial for systemic sclerosis is ongoing in the USA [31]. The study by Graziani et al. [32], shows that this medicine can inhibit the movement of the amoeboid cells.

Doxycycline (DOXY) has been approved by the Food and Drug Administration (FDA) for the prevention or treatment of specific infectious states, such as sexually transmitted infections, respiratory infections, bacterial infections, Lyme disease, eye infections, anthrax, acute intestinal amebiasis, traveler's diarrhea, and severe acne [33]. It has also been investigated for the treatment of specific tumors as some studies suggest that doxycycline can inhibit cell proliferation and migration, induce apoptosis and block the gap phase of cell cycle [34].

Fasudil (FAS) is the chemical substance that could have a positive effect on cell migration in terms of migrastatic treatment [35]. The possible mechanisms of FAS are improvement in coronary vasodilation, inhibition of apoptosis, and oxidative stress, inflammation relieving and reduction in endoplasmic reticulum and stress and metabolism reduction in endoplasmic reticulum [36].

According to the study by Stone et al. [37], the addition of the multitargeted kinase inhibitor midostaurin (MID) multitargeted kinase inhibitor to standard chemotherapy significantly prolonged overall and event-free survival among patients with acute myeloid leukemia and a FLT3 mutation. Clinical studies have investigated the utility of MID in solid tumors and lymphomas but have failed to replicate the preclinical findings [38].

Niclosamid (NICL) induces apoptosis by activating an intrinsic and caspase-independent pathway in human non-small cell lung tumor line A549 and CL1-5 cells [39]. Therefore, NICL is a potential candidate for anti-NSCLC therapy. Studies Lin et al. and Yeh et al. confirmed that this medicine inhibits a migration of cells.

Pimozide (PIM) - inhibited myofibroblast formation inhibition is assessed by the reduction of α-smooth muscle cell actin [40]. The study Dakir et al. [40] demonstrates the novel *in vitro* and *in vivo* antitumor activity of this medicine against breast and lung tumor cells and provides a proof of concept for the putative drug as a novel approach to anti-tumor therapy.

Migrastatics are becoming a novel category of antiinvasive and anti-metastatic drugs [3]. Putative candidates examined here are characterized in Table 1. Concentrations were determined according to recommended therapeutic plasma concentration when administering medicines.

**Table 1.** Putative migrastatics and their concentration

Putative migrastatics	Medicine (M) or Chemical (CH)	Concentration in PAMP	Reason for selection	Citation

4-Hydroxyacetophenone (4HAP)	CH	4 µM	Simple molecule that inhibits contractility	[30]
			inhibit amoeboid invasiveness in particular	[30]

Belumosudil (BEL)	M	1 µM	Newly approved ROCK II kinase inhibitor	[41]
			Inhibitor of amoebic invasiveness	[32]

Doxycycline (DOXY)	M	1 µg/ 1 ml	Inhibitor of mesenchymal invasiveness	[42]

Fasudil (FAS)	M and CH	10 µM	ROCK kinase inhibitor	[3]
			Inhibitor of amoebic invasiveness	[35]

Midostaurin (MID)	M	1 µM	Inhibitor mesenchymal invasiveness	[43]

Niclosamide (NICL)	M	1 µM	Effect on microtubules and a wide range of signaling important for migration (STAT3, mtor, Wnt)	[44]
			Potentially inhibit mesenchymal and amoeboid invasiveness	[45,46]

Pimozide (PIM)	M	10 µM	Arp2/3 complex inhibitor	[47]
			Inhibitor of mesenchymal invasiveness	
			Inhibits metastasis in an animal model	[40]

### 2.3 Coherence-controlled holographic microscopy

**Fig. 1.**
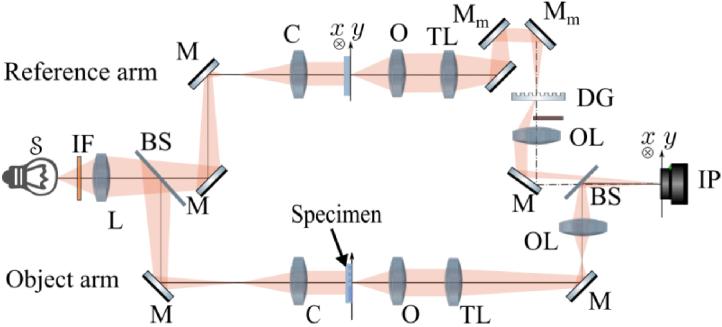
Optical setup of the coherence-controlled holographic microscope: S, light source; IF, interference filter; L, relay lens; BS, beam splitters; M, mirrors; Mm, movable mirrors; C, condensers; O, objective lenses; TL, tube lenses; DG, diffraction grating; OL, output lenses; IP, interference plane.

The optical setup of the Q-PHASE microscope (Fig. 1) is based on the Mach-Zehnder interferometer. It consists of two optical paths, the object and reference arm. These two paths contain optically equivalent microscope systems. The crucial functionality and benefits of this microscope depends on the diffraction grating (DG; transmission phase grating with groove frequency 150 mm−1, blazed at 760 nm for the first diffraction order) implemented in the reference arm according to principles proposed by Leith [48]. It guarantees an off-axis hologram formation in the interference plane (IP) for sources of an arbitrary degree of coherence. In our system, an LED was used for illumination to provide a spatially broad incoherent source, and the illuminating light was filtered using an interference filter (IF) with central wavelength of 660 nm and of 10 nm full width at half maximum to achieve also quasi-monochromatic illumination. The relay lens (L) images the source through a beam splitter (BS) to the front focal planes of the condensers (C; Nikon LWD condenser lenses, 0.52 NA, with adjustable aperture stop), forming Köhler illumination. The fundamental image properties depend on the parameters of condensers and objective lenses (O; Nikon Plan Fluorite Objectives, 10x/0.3 NA/16 mm WD) coupled with tube lenses (TL; Nikon, focal length 200 mm). The two-point resolution in the Q-PHASE microscope was close to the theoretical value 1.3 µm determined by the Airy-disc radius (see [12], sec. 6.2) of the microscope PSF for the above-mentioned experimental parameters. The microscope is very well automated and can be finely adjusted thanks to several stepper and piezo motors. The holograms were recorded in IP using the Andor Zyla 4.2 sCMOS camera.

### 2.4 Analysis of holographic incoherent quantitative phase imaging

For the hologram reconstruction and subsequent phase image analysis, we used the SophiQ software (TELIGHT Ltd. Brno, Czech Republic). The dry mass density *M* measured in pg/µm^2^ was calculated as

(1) $$M(x,y)=\frac{\varphi (x,y)\lambda }{2\pi \alpha },$$

where x,y are image pixel coordinates, and φ is the reconstructed phase in radians at that pixel. The wavelength λ of illumination light in our experiments was 0.66 µm and the specific refraction increment α is 0.18 µm^3^/pg [16].

The total mass of the cell is given as the sum of the dry mass density values determined from the phase at each pixel associated with a particular cell, such as

(2) $$Mass=\underset{N}{\sum }whM(i),$$

where *w* and *h* are pixel width and height respectively, *N* is the number of cell’s pixels and the summation goes from i=1 over all *N* pixels of the cell of interest. We also monitor the cell area defined as

(3) Area=Nwh,

Another parameter helping us evaluate morphological changes was the circularity percentage of individual cells:

(4) $$Circularity=100\frac{4\pi \times Area}{Perimeter^{2}}.$$

The perimeter length of the cell is also very informative parameter. For the perimeter of the cell, the software uses an iterative approach that follows the path of pixels at the edge of the cell.

For the analysis of cell movement, it is important to calculate the speed of migration:

(5) $$Speed(t_{n})=\frac{|WCG(t_{n})-WCG(t_{n-1})|}{t_{n}-t_{n-1}},$$

where $t_{n}$ is time at the moment *n* and $t_{n-1}$ is time at the previous moment n−1 *., WCG* (Weighted Center of Gravity) is a two-dimensional vector with x and y coordinates. It is another important parameter for subsequent analysis and it is defined as

(6) $$WCG=\frac{\sum_{N}whM(i)(x_{i},y_{i}),}{Mass}$$

where the meandering index is the curvature index of the cell movement.

(7) $$MeanderingI(t_{n})=\frac{|WCG(t_{n})-WCG(t_{0})|}{\sum_{1}^{n}|WCG(t_{n})-WCG(t_{n-1})|},$$

The numerator of the fraction (7) represents the Euclidean distance.

The SophiQ software provides more parameters, but these were chosen for the PAMP methodology. The entire classification procedure of the methodology was carried out in SophiQ, Microsoft Excel, and OriginPro programs.

### 2.5 PAMP methodology

Cells are cultivated at 60,000 cells/ml in Ibidi µ-Slide VI^0.4^ for 24 h in an incubator with 3.5% of CO_2_. Subsequently, the medium is replaced with a migrastatic agent at the given concentration listed in Table 1. Biocompatible silicone oil (Ibidi anti-evaporation oil) is added to the Ibidi µ-Slide VI^0.4^ reservoirs to prevent unwanted evaporation of the medium during the time-lapse recording. The reservoirs are closed with connectors (Elbow Luer Connector Male, Ibidi). The sample is inserted into the object arm of the Q-PHASE. The time-lapse recording was 20 hours (approximately the length of one cell cycle) at 5-minute intervals with Nikon Plan Fluorite Objective 10x/0.3 NA/16 mm WD. The Q-PHASE microscope is equipped with a motorized stage that enables multi-field time-lapse recording. The recording of multiple positions is thus a big advantage, e.g., in Ibidi µ-Slide VI^0.4^. After scanning one field of view, the microscope moves to another field of view in the examination chamber.

The Ibidi µ-Slide VI^0.4^ was used with all six examination channels. In this case, one channel was chosen as a control sample (CNT) and the other 5 contained medium with migrastatic. In one Ibidi, only one cell line was always found in all channels.

In the case of non-tumor NDHF cells, 2 Ibidi µ-Slide VI^0.4^ from one passage were examined simultaneously, therefore only one control is presented here, which applies to all migrastatics.

In the case of tumor cell lines A549 and HT1080, one Ibidi µ-Slide VI^0.4^ was measured each on different days, therefore two controls are presented here. Each control sample is for a given measurement. Recordings usually begin within one hour after the addition of the test substance.

### 2.6 PAMP construction

**Fig. 2.**
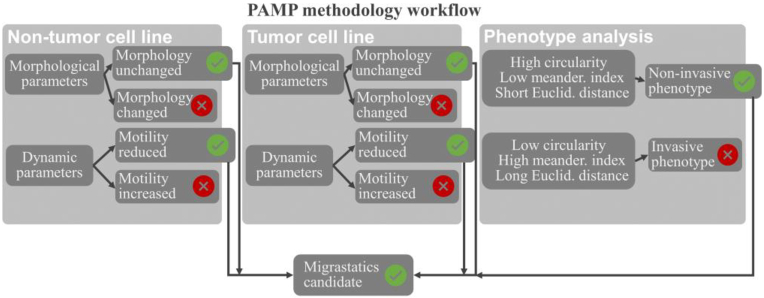
Diagram showing the PAMP methodology procedure. This concept consists of three parts (evaluation of non-tumor cells, tumor, and invasive phenotype). Subsequently, these parts are broken down, and a possible candidate for migrastatics is determined.

The PAMP methodology consists of three consecutive steps (Fig. 2). The aim of this methodology is to identify a migrastatic candidate (chemical or medicinal).

**Fig. 3.**
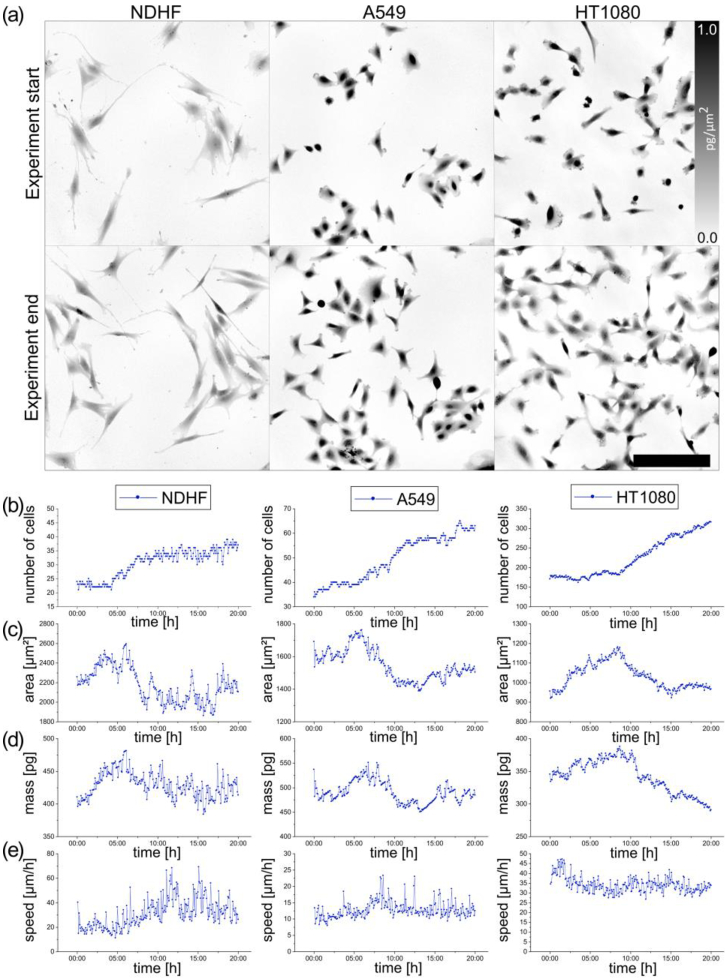
Example of the first step of the PAMP method ― the evaluation of non-tumor NDHF cells and A549 and HT1080 tumor cell lines in the control sample. (a) Images of QPI of NDHF, A549 and HT1080 cells in the control sample. Color bar in pg/µm^2^. Time-lapse QPI at the beginning and end of the measurement. Objective lens 10x/0.3. Scale bar 200 µm. (b) Time graph of number of cells. (c) Time graph of area. This parameter is calculated in µm^2^. Average values over time are shown. (d) Time graph of mass. This parameter is calculated in pg. Average values over time are shown. (e) Time graph of NDHF cell movement speed in [µm/h].

The first step is to evaluate the status of the control sample (CNT) in the non-tumor NDHF cell line. Individual time-lapse QPI of non-tumor cells (Fig. 3 (a)) can be displayed to detect the presence of cell death, infection, or other adverse effects. An important parameter for evaluating the condition of these cells is the increase in the number of cells (Fig. 3 (b)) in the control sample. If the number of cells in the control sample decreases, it can be assumed that the cells have been affected by some unwanted side effect and the migrastatic results may be affected too. Other parameters mass and area are shown in Fig. 3 c,d, help us to determine that the cells have stopped growing and this is not the purpose of migrastatics but of cytostatics, so we can say that the morphology changes. Growth arrest or cell death leads to an effect on the mass-area curve. The values of these parameters obtained at the beginning of the measurement should not be very different from the values at the end of the measurement.

In addition to the morphological changes in the behavior of the cells, it is also necessary to evaluate the dynamic properties of the cells in the control sample, especially the speed of migration, shown on the graph in Fig. 3 (e). In the control sample, the migration speed should be approximately constant. This control sample evaluation procedure also applies to tumor line A549 and HT1080 controls, shown in Fig. 3 (a - e). The analysis of these parameters is identical to the analysis of the effect of the migrastatic candidate and the values are compared with those of the control sample.

The second step is the evaluation of migrastatic potential in tumor cells (A549 and HT1080). The evaluation procedure is the same (morphological and dynamic changes in cell behavior) as for non-tumor NDHF cells.

The initial hypotheses for this study are that migrastatics should not affect the morphological behavior of non-tumor cells in any way, i.e. it is also undesirable for cell death or cell cycle arrest to occur, which can be seen not only in the area and mass graphs but also on other evaluated parameters such as roundness (a higher percentage than control) and densities (more pg/µm^2^). Dynamic changes and the speed of migration can be compared again with the control sample (Table 3). It is desirable that the migrastatic candidates reduce the speed of migration of cell movement.

Some potential migrastatics may have a late or early onset of action. Therefore, the results of the 20-hour time-lapse recording are divided into two equally long time periods.

The requirement is that migrastatic candidates preserve the average speed of migration of non-tumor cells while slowing down the migration of tumor cells and do not cause morphological changes signaling cytopathogenicity, particularly of non-tumor cells.

**Fig. 4.**
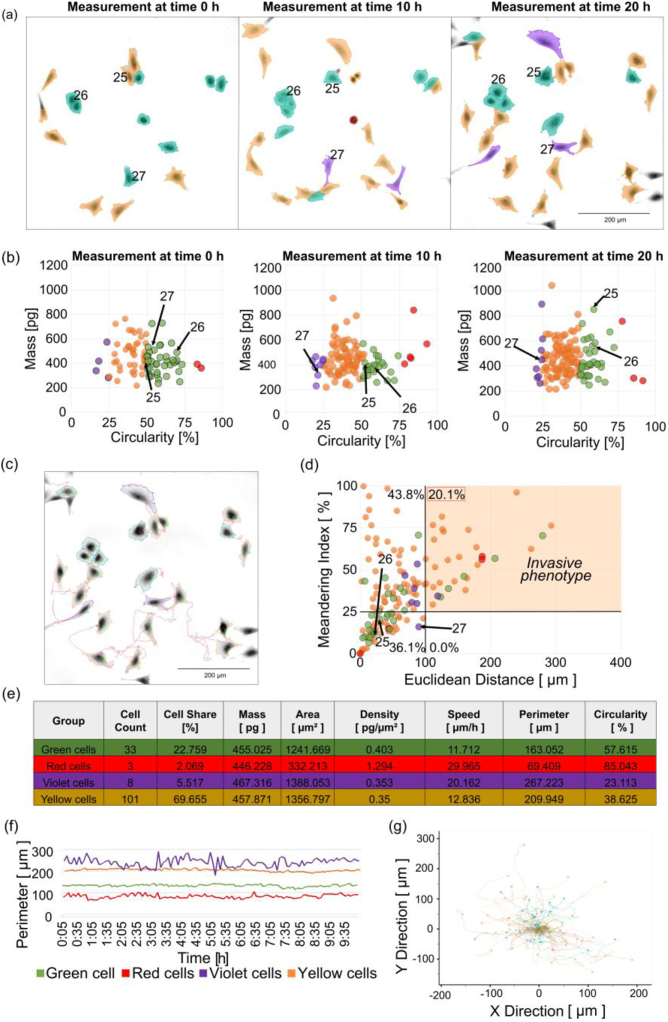
Sample of evaluating invasive phenotype of individual A549 tumor cells in the control sample. (a) Sample of QPI time-lapse of one scanned field of view at the start and end of the experiment. Objective lens 10x/0.3. Scale bar 200 µm. The color scale in pg/µm2. (b) Sample of feature plot of the circularity per mass at the start and end of the experiment. (c) Example of QPI images with trajectories of individual cells at the end of measurement. Trajectories are in µm. (d) Feature plot for the invasive phenotype. Graph of the ratio of Euclidean distance (x) and meandering index (y) with the division of cells into four quadrants. (e) The table of all parameters of individual groups of cells evaluates the state of the cells at the end of the measurement. (f) Time plot of the average perimeter values showing the time course of given groups of cells. The perimeter is in µm. (g) Migration rose plot showing individual cell trajectories. Individual tracks were shifted to the common origin. Axes are in µm.

The third step is to assess the invasive phenotype. This step relies on cell segmentation (Fig. 4 (a)) and subsequent cell division using mass (y) and circularity (x), see Fig. 4 (b). Cell values can be divided into four groups according to the circularity percentage (see Table 2). Each cell is assigned a color segmentation and an ID number during the analysis, which it had throughout the evaluation period. Trajectories of individual cells can also be evaluated, as shown in Fig. 4 (c).

**Table 2.** Separation of cells by percentage of circularity to assess and search for the invasive phenotype of cells

Color segmentation	Circularity [%]	Morphology	State of cell
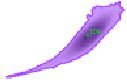	0-25	Very elongated	Anomalous or migratory cell – probably invasive phenotype
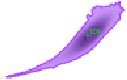	25-50	Elongated	Probably migratory
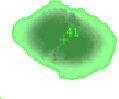	50-75	Oval	Resting state
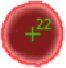	75-100	Rounded	Dividing cell or cell undergoing cell death

Subsequently, the cells are displayed on a graph of Euclidean distance (x) and meander index (y) elements, Fig. 4 (d). The percentage of invasive cells is in the upper right quadrant of this graph, i.e., Euclidean distance greater than 100 µm and meander index greater than 25%. The relationship between the meandering index and circularity, and the second selected is the location in the scatter plot defined by the meandering index (y) and the Euclidean distance (x). The purpose is to refine the classification of the invasive phenotype of tumor cells based on the position in the scatterplot. In the case of the invasive phenotype, the cell has a low percentage of circularity, a high meandering index, and high Euclidean distance value.

For a more accurate analysis of cells, it is possible to evaluate morphological and dynamic parameters for individual groups of cells using tables (Fig. 4 (e)), time graphs (Fig. 4(f)), and migration rose (Fig. 4 (g)). The color differentiation corresponds to the division according to the percentage of circularity from Table 2.

## 3. Results and discussion

### 3.1 Assessment of cell growth

**Fig. 5.**
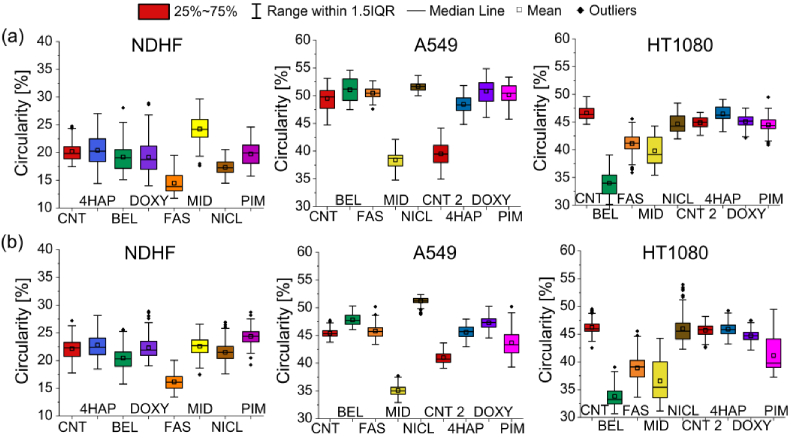
Boxplots of circularity illustrating responses of NDHF non-tumor cell line and A549 and HT1080 tumor cell lines to selected migrastatics. The line represents the medians, and the square represents the mean. (a) Box plots in the first 10 hours of measurement (00:00–10:00). (b) Box plots in the second 10 hours (10:05–20:00) of the measurement. Circularity is calculated in %. Each migrastatic has its own color in all graphs.

The Q-PHASE microscope defines the pattern of cell polarity by evaluating the morphological profile of migrating cells along with measuring their distribution in the nucleus and peripheral dry mass. From the QPI recordings, we evaluated the mean density (pg/µm^2^) and the circularity (%) of the cells. Circularity is evaluated in the same way as density. We are interested in the percentage of circularity, the higher circularity of more cells may indicate cell death. Which is undesirable in the case of migrastatic treatment. The analysis was divided into two time periods, the first 10 hours (Fig. 5 (a)) and the second 10 hours of measurement (Fig. 5 (b)).

**Fig. 6.**
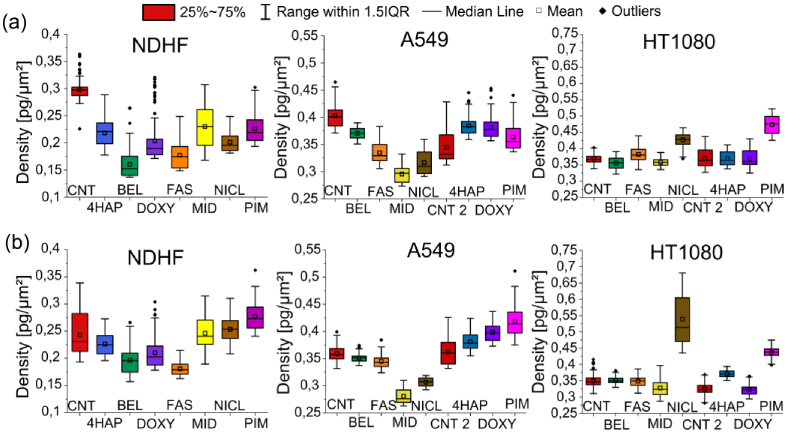
Boxplots of density illustrating responses of non-tumor NDHF cell line and A549 and HT1080 tumor cell lines to selected migrastatics. The line represents the medians, and the square represents the mean. (a) Box plots in the first 10 hours of measurement (00:00-10:00). (b) Box plots in the second 10 hours (10:05-20:00) of the measurement. The density is calculated in pg/µm^2^ as the mass per area of cells.

In this case, the density was calculated as the ratio of mass (pg) to area (µm^2^). The assessment was again divided into two time periods, the first 10 hours (Fig. 6 (a)) and the second 10 hours of measurement (Fig. 6 (b)). For a more accurate density analysis, the values in the time periods of 0, 10, and 20 hours are written in Table 3.

**Table 3.** Evaluation of NDHF, A549 and HT1080 cell lines density growth and decline in time courses

Cell line	Sample	00:00	10:00	Different between 00:00 and 10:00	20:00	Different between 10:00 and 20:00	Total time Different between 00:00 and 20:00
		Density [pg/µm^2^]	Density [pg/µm^2^]	Increase / decrease	Density [pg/µm^2^]	Increase / decrease	Increase / decrease
NDHF	CNT	0.29	0.30	+0.68%	0.20	-30.85%	-30.38%
	4HAP	0.18	0.22	+19.34%	0.27	+23.61%	+47.51%
	BEL	0.14	0.18	+26.95%	0.26	+44.13%	+82.98%
	DOXY	0.19	0.18	-6.81%	0.27	+51.12%	+40.84%
	FAS	0.15	0.17	+12,08%	0.18	+10.18%	+23.49%
	MID	0.17	0.27	+58,93%	0.20	-26.97%	+16.07%
	NICL	0.19	0.21	+12,17%	0.26	+21.23%	+35.98%
	PIM	0.24	0.30	+23,75%	0.25	-17.51%	+2.08%
A549	CNT	0.38	0.39	+0.79%	0.36	-5.71%	-4.97%
	BEL	0.37	0.36	-4.28%	0.36	+0.28%	-4.01%
	FAS	0.36	0.38	+4.75%	0.34	-10.67%	-6.43%
	MID	0.32	0.30	-5.98%	0.27	-11.04%	-16.35%
	NICL	0.36	0.29	-18.61%	0.32	+8.19%	-11.94%
	CNT 2	0.34	0.43	+27.38%	0.35	-18.93%	+3.27%
	4HAP	0.37	0.39	+5.69%	0.39	0%	+5.69%
	DOXY	0.37	0.41	+11.59%	0.39	-4.83%	+6.20%
	PIM	0.35	0.38	+6.53%	0.41	+10.4%	+17.61%
HT1080	CNT	0.40	0.40	+0.75%	0.32	-21.84%	-21.25%
	BEL	0.37	0.35	-4.34%	0.38	+7.65%	+2.98%
	FAS	0.34	0.37	+9.23%	0.32	-12.26%	-4.17%
	MID	0.37	0.39	+4.62%	0.32	-17.14%	-13.32%
	NICL	0.37	0.44	+18.97%	0.68	+55.13%	+84.55%
	CNT 2	0.40	0.34	-8.13%	0.30	-12,68%	-28.50%
	4HAP	0.40	0.35	-11.00%	0.33	+2.87%	-8.44%
	DOXY	0.41	0.33	-19.61%	0.30	-9.76%	-27.45%
	PIM	0.44	0.43	-2.27%	0.4	-6.98%	-9.09%

In Fig. 5 and 6 and Table 3 for both tumor cell lines A549 and HT1080, the control sample CNT 1 was bound to BEL, FAST, MID, and NICL, and the control sample CNT 2 was bound to 4HAP, DOXY, and PIM in order not to combine the two different measurements obtained on different days.

The results of the PAMP method in these non-tumor cells show that MID and BEL affected density the most. Circularity was most affected by MID (increase) in the first time period of measurement. The lowest circularity was recorded for FAS.

We evaluated boxplots of density (pg/µm^2^) and circularity (%) of tumor cells from QPI recordings. In this case, the density was calculated as the ratio of mass (pg) to area (µm^2^) and circularity in %.

Graphs of density and circularity show that a MID was able to reduce the speed of cell movement of A549 and HT1080 and did not cause changes in morphological behavior. But the changes caused NICL.

From Tab. 3 it is evident that the most important increase in the density of dry mass of cells (pg/µm^2^) for non-tumor NDHF cells was caused by BEL, for tumor cells A549 was PIM and HT1080 of NICL.

### 3.2 Detection of signs of pathogenicity

**Fig. 7.**
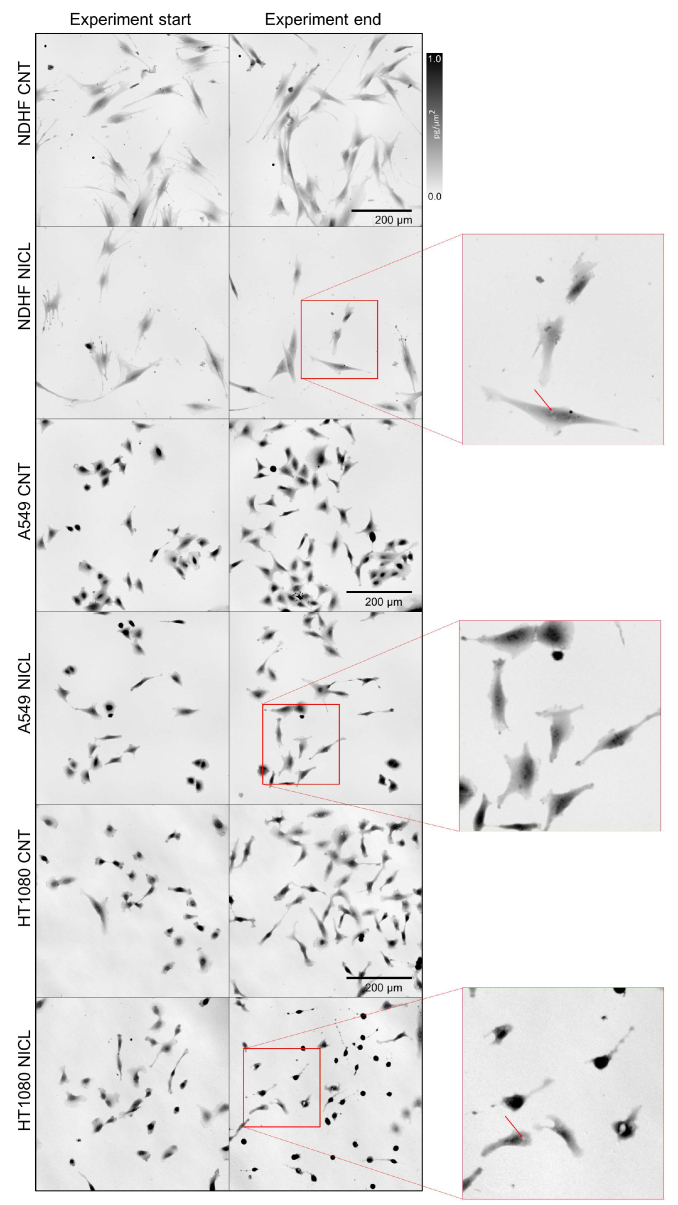
Examples of QPI images picked up from time-lapse recordings of NDHF, A549 and HT1080 cells show morphological changes induced by niclosamide. In magnified areas from the red squares the red arrows indicate cytoplasmic vacuolation suggesting apparently un endoplasmic reticulum stress. Objective lens 10x/0.3. Scale bar 200 µm.

The most common pathological phenomenon detectable *in vitro* is the increase in circularity percentage that can mark an impending cell death (apoptosis or necrosis). Similar warning brings to attention changes around the nucleus, such as cytoplasmic vacuolation after endoplasmic reticulum stress [49] seen in the NDHF cells after niclosamide application (Fig. 7).

### 3.3 Assessment of the migratory reaction

#### 3.3.1 Assessment of the migratory reaction of the non-tumor NDHF line

After 20 hours, cells in control conditions maintained their physiological state, while cells exposed to migrastatics responded to the given environment. The speed of migration classification was first performed on control non-tumor NDHF cells. The same procedure was then repeated for the tumor lines. The classification is based on the evaluation of the methodology based on the QPI time-lapse.

The changes in the speed of cell migration that result from the implementation of the approach based on the evaluation of two time periods are summarized in Table 4.

**Table 4.** Evaluation of cell migration speed values in non-tumor NDHF

Cell line	Sample	Time	Mean [µm/h]	Median [µm/h]	Speed [%]	P-value
NDHF	CNT	00:05-10:00	24.4 ± 8.6	22.6	-	-
		10:05-20:00	38.6 ± 11.2	35.7	-	-
	4HAP	00:05-10:00	33.2 ± 9.9	32.9	↑ 36.1	***
		10:05-20:00	36.7 ± 10.5	34.4	↓ 5.1	n. s.
	BEL	00:05-10:00	19.5 ± 10.5	15.7	↓ 20.2	***
		10:05-20:00	37.2 ± 11.9	34.0	↓ 3.8	n. s.
	DOXY	00:05-10:00	31.5 ± 9.5	29.1	↑ 29.2	***
		10:05-20:00	36.0 ± 7.6	34.3	↓ 6.9	*
	FAS	00:05-10:00	32.5 ± 12.8	30.5	↑ 33.3	***
		10:05-20:00	32.1 ± 10.0	30.0	↓ 16.9	***
	MID	00:05-10:00	35.4 ± 11.2	33.4	↑ 45.2	***
		10:05-20:00	29.5 ± 10.6	27.7	↓ 23.7	***
	NICL	00:05-10:00	20.6 ± 9.4	16.9	↓ 15.4	**
		10:05-20:00	26.2 ± 9.5	24.3	↓ 32.3	***
	PIM	00:05-10:00	28.8 ± 7.8	27.8	↑ 18.2	***
		10:05-20:00	36.8 ± 9.2	35.0	↓ 4.8	n. s.

The median here represents the middle value of all in a given time period and is used for comparison with the mean of the migration speed. The cell migration speed as a percentage was calculated as a percentage of the ratio of the speed of the given medicines to the control.

Overall accuracy was determined by the p-value of the two-sample F-test for Variance for the need to test the equality of sample variance. Followed by the T-Test: Two-Sample Assuming Equal or Unequal Variances according to p-value ± 0.05 from F-Test for Variance. The two-tail P-value corresponds to the T-Test. Symbols indicating significance are placed in the table: * represents P < 0.05, ** P < 0.01, *** P < 0.001, and n. s. is for not significant.

The results from this table indicate that BEL and NICL were able to reduce the speed of migration at both time points.

#### 3.3.2 Assessment of the migratory reaction of the tumor line A549 and HT1080

The values for the evaluation of the tumor cell lines migration speed (Table 5) were also calculated using the same methods in the case of non-tumor cells. Thus, for both tumor cell lines, the CNT control sample was bound to BEL, FAST, MID, and NICL, and the CNT2 control sample was bound to 4HAP, DOXY, and PIM in order not to combine two different measurements obtained on different days.

**Table 5.** Evaluation of cell migration speed values in tumor cell lines A549 and HT1080

Cell line	Sample	Time	Mean [µm/h]	Median [µm/h]	Speed [%]	P-value
A549	CNT	00:05-10:00	12.7 ± 2.9	12.0	-	-
		10:05-20:00	13.0 ± 2.3	12.4	-	-
	BEL	00:05-10:00	11.9 ± 2.5	11.3	↓ 6.6	*
		10:05-20:00	11.6 ± 2.2	11.2	↓ 10.8	***
	FAS	00:05-10:00	13.1 ± 2.4	12.5	↑ 2.7	n. s.
		10:05-20:00	14.5 ± 2.2	14.1	↑ 11.8	***
	MID	00:05-10:00	10.6 ± 2.3	10.1	↓ 6.9	***
		10:05-20:00	10.6 ± 3.7	9.5	↓ 18.1	***
	NICL	00:05-10:00	5.1 ± 1.6	4.9	↓ 60.0	***
		10:05-20:00	5.7 ± 1.9	5.3	↓ 56.3	***
	CNT 2	00:05-10:00	14.3 ± 3.5	13.9	-	-
		10:05-20:00	13.8 ± 3.1	13.1	-	-
	4HAP	00:05-10:00	15.3 ± 3.7	14.5	↑ 6.6	*
		10:05-20:00	14.8 ± 3.4	14.0	↑ 7.3	*
	DOXY	00:05-10:00	13.6 ± 3.5	13.1	↓ 5.4	n. s.
		10:05-20:00	14.9 ± 3.1	14.1	↑ 7.9	***
	PIM	00:05-10:00	9.1 ± 2.2	8.9	↓ 36.4	***
		10:05-20:00	14.9 ± 4.6	13.6	↑ 7.8	*
HT1080	CNT	00:05-10:00	34.9 ± 4.7	34.0	-	-
		10:05-20:00	33.4 ± 2.9	33.4	-	-
	BEL	00:05-10:00	36.1 ± 3.7	35.8	↑ 3.3	*
		10:05-20:00	33.5 ± 3.2	33.3	↑ 0.3	n. s.
	FAS	00:05-10:00	56.8 ± 5.9	56.6	↑ 62.6	***
		10:05-20:00	49.2 ± 4.7	48.9	↑ 47.2	***
	MID	00:05-10:00	24.6 ± 5.2	23.5	↓ 29.5	***
		10:05-20:00	22.0 ± 3.1	22.0	↓ 34.0	***
	NICL	00:05-10:00	32.5 ± 4.3	32.6	↓ 7.0	***
		10:05-20:00	14.3 ± 5.1	14.0	↓ 57.3	***
	CNT 2	00:05-10:00	28.9 ± 4.5	28.2	-	-
		10:05-20:00	30.2 ± 3.6	30.2	-	-
	4HAP	00:05-10:00	31.1 ± 3.8	31.3	↑ 7.6	***
		10:05-20:00	31.9 ± 3.3	31.6	↑ 5.8	***
	DOXY	00:05-10:00	28.7 ± 4.5	28.4	↓ 0.7	n. s.
		10:05-20:00	32.0 ± 4.4	32.4	↑ 6.1	***
	PIM	00:05-10:00	35.4 ± 6.5	34.3	↑22.2	***
		10:05-20:00	30.8 ± 3.3	30.9	↑ 2.2	n. s.

The median here represents the middle value of all in a given time period and is used for comparison with the mean of the migration speed. The cell migration speed as a percentage was calculated as a percentage of the ratio of the speed of the given medicines to the control.

Symbols indicating significance are placed in the Table 5: * represents P < 0.05, ** P < 0.01, *** P < 0.001, and n. s. is for not significant results. The median expressed as a percentage was calculated as a percentage between the ratio of the median and the mean. The cell translocation expressed as a percentage was calculated as a percentage of the ratio of the given drug to the control.

Results have shown that for the A549, only BEL, MID, and NICL were able to reduce the speed of migration. In the case of HT1080 cell line the migration speed was reduced only with MID and NICL.

### 3.4 Invasive phenotype

The PAMP method evaluates the degree of tumor cells invasiveness after the application of drugs. Table 6 shows the percentage of cells that are still invasive even after the application of migrastatics; more than 100 µm according to the Euclidean distance and their Meandering index was higher than 25%.

**Table 6.** Expression table of the invasive phenotype of NDHF, A549 and HT080 cells after 20 hours of migrastatics treatment

Sample		Invasive phenotype [%]	
		10 h	20 h
NDHF	Control sample	38.3	22.5
	4-hydroxyacetophenone	25	26.9
	Belumosudil	36	34.1
	Doxycycline	39.3	27.8
	Fasudil	45.1	37.3
	Midostaurin	34.1	31.7
	Niclosamide	48.8	35
	Pimozide	30	10
Sample		Invasive phenotype [%]	
		**10 h**	20 h
A549	Control sample 1	16.2	29
	Belumosudil	13.3	20
	Fasudil	12	21.3
	Midostaurin	9.2	14.2
	Niclosamide	0	8.8
	Control sample 2	25.1	38.5
	4-hydroxyacetophenone	22.5	37.4
	Doxycycline	15.3	29.9
	Pimozide	28.6	41.2
Sample		Invasive phenotype [%]	
		10 h	20 h
HT1080	Control sample 1	32.7	30.7
	Belumosudil	38.9	30.3
	Fasudil	34.5	29.3
	Midostaurin	37.5	37.4
	Niclosamide	32.9	34.5
	Control sample 2	36.4	40.8
	4-hydroxyacetophenone	36.6	34.2
	Doxycycline	34.2	31.9
	Pimozide	41.7	32.5

The results show that in the case of A549 cells, NICL most affected tumor cells. Conversely, after 4HAP, DOXY, and PIM there was no significant reduction in the percentage of invasive cells. In HT1080 cells, NICL did not have as strong effect as in the case of A549 cells. Only PIM achieved the greatest reduction.

## 4. Conclusion

We have developed the Primary Assessment of Migrastatic Potential (PAMP) *in vitro* methodology for rapid introductory evaluation of the putative migrastatics potency. This method was designed to measure a reduction of adherent cells random migration in 2D culture conditions while registering cell physiology undisturbed.

In the A549 cells, BEL, MID and NICL reduced the speed speed, BEL and NICL influenced moreover the morphology, especially the high percentage of circularity and low density. BEL and MID did not change the number of cells with an invasive phenotype when compared to the control sample. NICL was also able to reduce the number of cells with an invasive-like morphology compared to the control sample. We recommend considering MID as a possible candidate for interfering with migration. BEL and NICL require finding a suitable concentration (lower) that will still negatively affect the speed of migration but not the morphology.

In HT1080 cells, cell migration rates were reduced after applying MID and NICL. MID did not cause morphological changes, but NICL caused an increase in density when compared to the control sample and high circularity of cells. The number of invasive cells after the MID and NICL application did not change significantly during the measurement. Nevertheless, we also recommend MID as a possible migrastatic candidate and NICL drug for additional testing with lower concentrations.

The primary assessment of migrastatic potential (PAMP) is based on selecting suitable tumor cell lines for the multifield time-lapse recording QPI follow-up of the anti-migratory effect of the tested medicines. Such an approach was made possible by exploiting the capability of QPI obtained from Q-PHASE microscope for precise non-invasive imaging of cells and measuring their dry mass. Based on the results of this pilot experiment we suggest midostaurin as a potential migrastatic for further preclinical validation. We have shown that QPI is a reliable and economical microscopical technology for the *in vitro* examination of dynamics of cells migratory behaviour induced by potential migrastatics.

## Data Availability

Data underlying the results presented in this paper are not publicly available at this time but may be obtained from the authors upon reasonable request.
